# Hurdle Clearance Detection and Spatiotemporal Analysis in 400 Meters Hurdles Races Using Shoe-Mounted Magnetic and Inertial Sensors

**DOI:** 10.3390/s20020354

**Published:** 2020-01-08

**Authors:** Mathieu Falbriard, Maurice Mohr, Kamiar Aminian

**Affiliations:** 1 Laboratory of Movement Analysis and Measurement, EPFL, 1015 Lausanne, Switzerland; 2 Institute of Sport Science, University of Innsbruck, 6020 Innsbruck, Austria; maurice.mohr@uibk.ac.at (M.M.); kamiar.aminian@epfl.ch (K.A.)

**Keywords:** inertial sensors, 400 m hurdles, magnetometer, hurdle clearance, IMU, flight time, speed

## Abstract

This research aimed to determine whether: (1) shoe-worn magnetic and inertial sensors can be used to detect hurdle clearance and identify the leading leg in 400-m hurdles, and (2) to provide an analysis of the hurdlers’ spatiotemporal parameters in the intervals defined by the hurdles’ position. The data set is composed of MIMU recordings of 15 athletes in a competitive environment. The results show that the method based on the duration of the flight phase was able to detect hurdle clearance and identify the leading leg with 100% accuracy. Moreover, by combining the swing phase duration with the orientation of the foot, we achieved, in unipedal configuration, 100% accuracy in hurdle clearance detection, and 99.7% accuracy in the identification of the leading leg. Finally, this study provides statistical evidence that contact time significantly increases, while speed and step frequency significantly decrease with time during 400 m hurdle races.

## 1. Introduction

The last decade has seen a growing trend towards magnetic inertial measurement units (MIMU)-based studies in track and field races, with the majority focusing on sprint distances. These studies differ in terms of sensor configuration, sensor location, and type of parameter measured [1]. Several groups have used inertial sensors in sprint running to characterize temporal parameters [2,3,4], body-segment orientation [5,6], ground reaction forces [7,8], and speed [9,10,11]. Surprisingly, only a few studies used MIMU to quantify spatiotemporal parameters in hurdle races. Recently Ho, Chang and Lin [12] used high-speed video cameras and inertial sensors strapped on the dorsal surface of each foot to analyze flight time, hurdle cycle time (i.e., the time between hurdles) and hurdle cycle velocity (i.e., hurdle cycle time divided by the distance between hurdles) in 110-m hurdles. Unfortunately, the authors offered no explanation about the method employed to detect the time point of hurdle clearance (HC) or how they measured the parameters above. The authors in [13] used inertial measurement units (IMU) to evaluate the kinematics of the hurdlers’ upper limbs and reported the linear velocities and the trajectory of the segments during hurdle clearance. Overall, little research has been performed specifically on 400-m hurdles [14], and no wearable system has been proposed to detect HC and identify the leading leg (LL), i.e., the leg attacking the hurdle.

The variations in the average speed, contact time, flight time, and step frequency in between the hurdles and the side of the leading leg are all relevant indicators of the athletes’ racing strategy, and thus can have a significant impact on performance. Currently, such analysis requires a set-up with multiple video cameras around the track and time-consuming manual post-processing of the data. A wearable system capable of providing instant feedback would significantly improve our capacity to monitor the training status and performance of athletes.

In alpine skiing, gate crossings have been detected through the use of magnets placed into the snow and a magnetometer worn by the athlete [15]. Although potentially transferable and useful, this technique has not yet been tested to measure the timing of hurdle crossings. Therefore, the primary aim of this study was to propose and test different methods based on foot-worn MIMU to detect HC and identify the LL. Furthermore, magnets fixed on the hurdles were tested as a complementary method to detect HC. As a secondary aim of this study, the changes throughout the race of contact time, flight time, running speed, and step frequency were analyzed to explore the relationship between the athlete’s caliber and racing strategy.

## 2. Materials and Methods

### 2.1. Protocol

**Figure 1:**
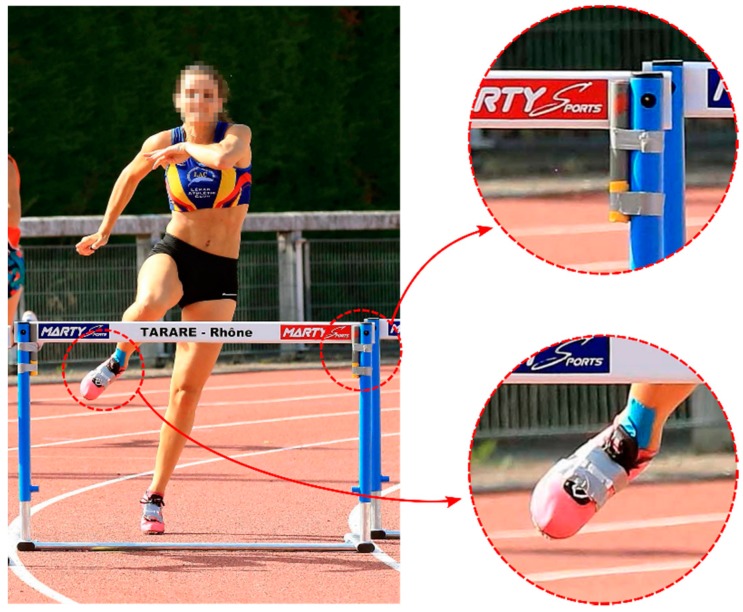
One participant clearing a hurdle with two shoe-mounted IMUs and one magnet bar on each of the vertical poles of the hurdle.

In this study, 16 athletes (n = 10 males (age: 22 ± 4 years, height: 183 ± 2 cm, weight: 69 ± 6 kg, time: 57 ± 3 s), n = 6 females (age: 23 ± 3 years, height: 165 ± 4 cm, weight: 55 ± 2 kg, time: 64 ± 3 s)) volunteered to perform one 400 m hurdles race equipped with IMUs. The measurement took place during an outdoor competition with participants aiming for a qualification, thus running at their maximum speed. The athletes were equipped before the warm-up session to not disturb their preparation routine, and the sensors were collected at the end of the race. Moreover, each of the 10 hurdles in the second lane was equipped with two magnet bars (Figure 1). In 400 m hurdling, the distance (*D_H_*) in between two hurdles is 35 m. The distance (*D_H_*) between the starting line and the first hurdle is 45 m, and the distance between the last hurdle and the finish line is 40 m, hence *D_H_* = {45, 35, …, 35, 40} with dim(*D_H_*) = 11 intervals. The study was conducted in accordance with the Declaration of Helsinki, and the protocol was approved by the local Ethics Committee. All subjects gave their written informed consent for inclusion in the study.

### 2.2. Instrumentation

Each participant was equipped with one shoe-mounted inertial measurement unit (IMU) (Physilog4, Gait Up SA, Lausanne, Switzerland, weight: 19 g, size: 50 × 37 × 9.2 mm) affixed on the dorsum of the foot with adhesive tape (Figure 1).

The left and right foot IMUs were synchronized using radio frequencies. The configuration included an accelerometer at 500 Hz (±16 g operating range), a gyroscope at 500 Hz (±2000 °/s operating range) and a magnetometer at 71Hz (±1000 μT operating range). The magnets were constructed by vertically stacking 8 small neodymium magnets (S-20-10-N, Supermagnete, Uster, Switzerland) spaced by 5 mm into a 12 cm long stick. The magnets were fixed on each side of the hurdle at the top of the vertical poles (Figure 1). We aimed for the magnets to be as close of possible to the foot-worn magnetometers when passing over the hurdle. Finally, the video of each race was recorded at 25 frames/s and used for verification purposes in this study (leading leg identification and the number of steps per interval manually labeled).

All the subsequent data processing tasks described in this manuscript, the implementation of the HC and LL detection algorithms, and the analysis of the results were performed using the MATLAB software (R2018b, MathWorks, Natick, MA USA) and required no external libraries.

### 2.3. Data Processing

#### 2.3.1. Preprocessing, Calibration, and Segmentation

**Figure 2:**
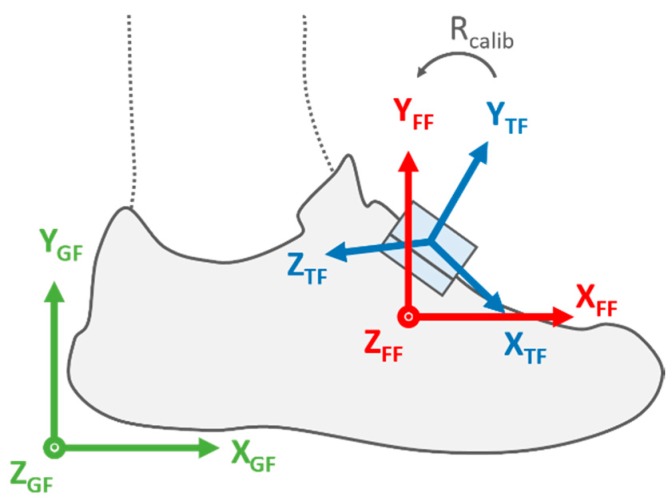
The orientation of the IMU technical frame (blue), the foot functional frame (red) and global frame (green) when standing. *R_calib_* is the rotation matrix obtained by the functional calibration.

The accelerometer and gyroscope sensors were calibrated, as described in [16]. The magnetometer offset, sensitivity, and axis-misalignment were corrected using the method proposed in [17] with calibration data recorded on-site the day of the event. The angular velocity and acceleration signals were low-pass filtered using a 2nd-order Butterworth filter with a cut-off frequency at 70 Hz. Functional calibration of the IMUs was performed as described in [18]: we used a standing period to define the functional frame (FF) vertical axis, the first component resulting from the principal component analysis (PCA) of the angular velocity during running to define the mediolateral axis of the foot, and we set the anterior-posterior axis orthogonally to the first two (Figure 2). The accelerometer, gyroscope, and magnetometer data were then expressed in the FF.

We defined the start of the race as the time *t_start_*, which occurred 200 ms before the first manually detected acceleration peak measured when the athlete was still in the starting blocks. The 200 ms offset corresponds to the estimated response-time of the participants [19], and the acceleration peak to the instant when the athlete starts pushing on the starting blocks. The races were then segmented using *t_start_* and the official race time (*T_race_*) of the participants.

#### 2.3.2. Temporal Analysis and Orientation Estimation

The stepwise temporal analysis was carried out as in [18] with minor adaptations to improve the robustness of event detection (i.e., mid-swings, initial contact, and terminal contact). The performance of the detection algorithm was indeed affected by the noise generated by the hurdle clearance movements, the adaptation steps occurring before and after the hurdle, and by the high running speeds. Since initial (IC) and terminal contact (TC) generate high-frequency oscillations in the acceleration signal, we restrained the search window for IC and TC events using the envelope of the signal. We computed the envelope using two successive wavelet transforms. First, we applied a high-pass filter (fc = 100 Hz) on the acceleration norm, which preserved only the high-frequency oscillations at IC and TC. We then rectified the signal and applied a low-pass filter (fc = 5 Hz). Although the successive filters resulted in a low amplitude signal, the shape of the envelope preserved two peaks where the high-frequency oscillations of IC and TC occurred. Features detection within these IC and TC limits was carried out as in [18], and the detection results of each trial were visually inspected to ensure that the algorithm correctly detected IC, TC, and mid-stance (MS) at each step. Note that MS corresponds to the event where the angular velocity in the sagittal plane of the foot is minimum.

We obtained the 3D orientation of the foot using strap-down integration [20] and a drift correction method based on the assumption that the global frame (GF) and the FF were aligned at MS (Figure 2). Hence, the orientation of the foot between two successive strides *i* and *i* + 1 was computed in the GF set at *MS*(*i*). Furthermore, the inclination of the foot in the starting-blocs was found using the orientation of the gravitational acceleration in the FF. Two Euler angles were extracted from the quaternion notation in the ZYX order: (1) the pitch angle (*θ*) defined as the rotation in the sagittal plane, and (2) the yaw angle (*ψ*) defined as the rotation in the horizontal plane.

### 2.4. Hurdle Clearance Detection

**Figure 3:**
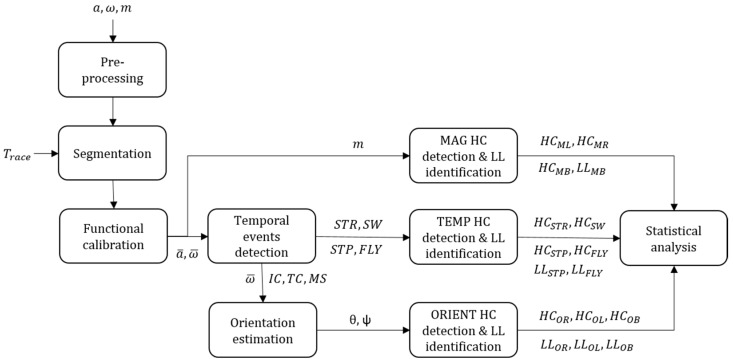
Flow chart of the proposed hurdle clearance detection method. We defined the inputs as follows: *a* the accelerometer data, ω the gyroscope data, *m* the magnetometer data, *T_race_* the official race duration, *θ* the pitch angle, and *ψ* the yaw angle of the foot. Initial contact (IC), terminal contact (TC), mid-stance (MS), stride time (STR), swing phase duration (SW), step duration (STP), and flight phase duration (FLY) result from the temporal analysis. Hurdle clearance (HC) detection results are shown as *HC_XX_* and leading leg detection results as *LL_XX_*, where XX describes the detection method.

Three methods have been implemented to detect HC and identify LL; (1) MAG: using the magnetometer signal, (2) TEMP: using the temporal events, and (3) ORIENT: using the foot orientation (i.e., pitch and yaw angles). For each of these methods, both a unipedal (i.e., one foot-worn IMU) and a bipedal (i.e., one IMU on each foot) configuration were tested. Each method was developed independently of the two others and used different parameters. Because the total number of hurdles (*N_hurdles_*) and the distance between the hurdles (*D_H_*) were fixed, these parameters were considered as inputs of the system. The general flow chart of the methods is described in Figure 3.

#### 2.4.1. MAG: Magnets and Magnetometer Based Detection

**Figure 4:**
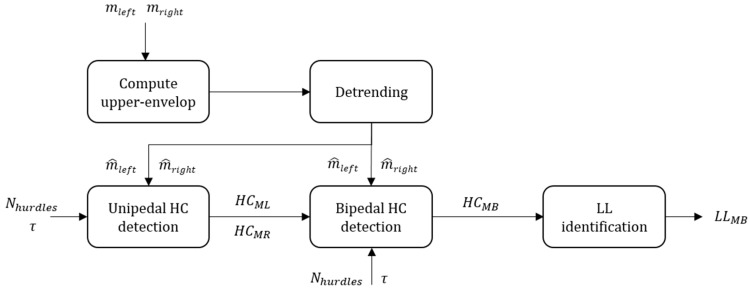
Block diagram of the MAG method. In this figure, *m_left_* and *m_right_* represent the magnetometer norm of the left and right foot, *N_hurdles_* the number of hurdles to detect and *τ* the minimum time difference between two consecutive HC.

This method assumes that the two magnets affixed on each side of the hurdle (Figure 1) locally increased the magnitude of the magnetic field. The HC detection, therefore, consisted of finding peaks on the filtered magnetometer norm (Figure 4).

We computed the upper envelope of the magnetometer norm using spline interpolation over local maxima separated by at least 0.5 s (maximum step frequency reported in [21]). The envelop signal was then normalized by its mean to facilitate the comparison of the peak absolute values between the two feet. Unipedal HC detection involved finding the *N_hurdles_* highest peaks separated by at least *τ* second (Equation (1)) on ${\hat{m}}_{right}$ and ${\hat{m}}_{left}$ for the right and left leg, respectively:

(1) $$\tau =\min\left(D_{H}\right)/V_{max}$$

where *D_H_* is the set of interval length and *V_max_*, the maximum running speed considered. As min(*D_H_*) = 35 m and *V_max_* was set at 11.67 m/s (42 km/h), which is slightly faster than the average speed of the current 100 m sprint World Record, *τ* was set at 3 s.

The times of the *N_hurdles_* highest peaks were then labeled as *HC_ML_* and *HC_MR_* for the left and right leg, respectively, with dim(*HC_ML_*) = dim(*HC_MR_*) = *N_hurdles_*. We obtained the bipedal detection results, namely *HC_MB_*, by combining *HC_ML_* and *HC_MR_* using to the following rules:

(1) If |*HC_ML_*(*i*) − *HC_MR_*(*j*)| < 0.4 s, *i* and *j* ∈ {1, …, *N_hurdles_*}, then 0.5 * (*HC_ML_*(*i*) + *HC_MR_*(*j*)) was added to HCMB. Here, we assumed that if two HC events occurred within a short period (i.e., 0.4 s = average flight time in [22]) and were detected on the left and right foot distinctively, then these events were likely to correspond to a true HC. As we could not predict which of the left or right event was more accurate, we defined the time of the true HC event as the average of the left and right foot events.
(2) The *i* and *j* indices not considered in step 1 were recursively added to HCMB until dim(HCMB) = *N_hurdles_*. The greatest peaks were added first if they were minimum *τ* = 3 s away from all the HC already in HCMB. Finally, the results were sorted in their order of appearance within the race.

Leading leg identification was only possible for the bipedal detection (*LL_MB_*), where we assumed that the leg with the earliest magnetic peak was the LL.

#### 2.4.2. TEMP: Temporal Event-Based Detection

This method supposes that the HC strides have longer phase durations in comparison to regular running strides. The phases considered were stride time (STR), swing phase duration (SW), step duration (STP), and flight phase duration (FLY) with the time series estimated as in Equations (2)–(5):

(2) STR(i)=IC(i+2)−IC(i)

(3) SW(i)=IC(i+2)−TC(i)

(4) STP(i)=IC(i+1)−IC(i)

(5) FLY(i)=IC(i+1)−TC(i)

Note that the estimation of these four temporal parameters required the detection of different events and necessitated different sensor configurations (Table 1). Since IC is more precisely detected than TC in running [18], we decided to keep STR and STP in the analysis, although SW and FLY offer narrower windows for HC detection (i.e., SW and FLY occur within STR and SPT, respectively). Moreover, STP and FLY parameters both require a bipedal configuration while STR and SW can be estimated from a single IMU.

To remove the trend induced by fatigue [21], we subtracted the moving average from the STR, SW, STP, and FLY time series using a window of length *K* (*K* equal to 60 steps for STP and FLY and 30 strides for STR and SW). For each parameter, the indices of the *N_hurdles_* highest peaks (i.e., the longest phase durations), separated by at least *τ* =3 s, were defined as i_k_ where *k* = 1 … *N_hurdles_*. Equations (6)–(9) show how the exact times of the HC were obtained based on the selected i_k_ periods of each parameter:

(6) $$HC_{STR}\left(k\right)=0.5\times \left(IC\left(i_{k}\right)+IC\left(i_{k}+2\right)\right)$$

(7) $$HC_{SW}\left(k\right)=0.5\times \left(TC\left(i_{k}\right)+IC\left(i_{k}+2\right)\right)$$

(8) $$HC_{STP}\left(k\right)=IC\left(i_{k}+1\right)+0.74\times \;\left(IC\left(i_{k}+2\right)-IC\left(i_{k}+1\right)\;\right)$$

(9) $$HC_{FLY}\left(k\right)=TC\left(i_{k}+1\right)+0.65\times \;\left(IC\left(i_{k}+2\right)-TC\left(i_{k}+1\right)\;\right)$$

**Figure 5:**
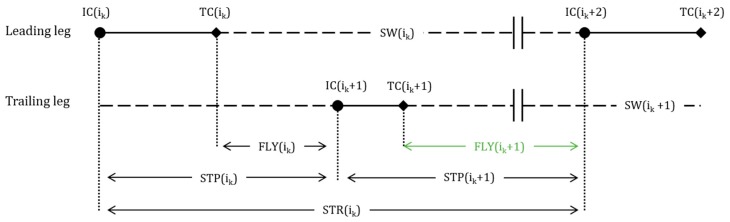
A sequence of temporal events for the leading and trailing leg. IC events are shown with circles, TC events with diamonds. HC events are shown with two parallel vertical bars, ground contact with a solid horizontal line, and SW with a horizontal dashed line. In green, the flight phase within which HC detected from MAG, TEMP, and ORIENT method would be classified as correctly detected.

In the unipedal cases (Equations (6) and (7)), we used a 0.5 factor because the exact time point of the HC event depends on the location of the IMU (i.e., on the leading leg or the trailing leg) (Figure 5). In Equations (8) and (9), the 0.74 and 0.65 coefficients were based on the results of previous research [23,24,25]. In these studies, the last ground contact time before HC lasted for approximately 25% of the total step duration. Furthermore, hurdle clearance occurred after 65% of the total HC distance, so if the speed is considered constant, 65% of flight time. The 0.74 factor of Equation (8) was found using the two coefficients mentioned above (Equation (10)):

(10) 0.74=round(0.25+(0.65×(1−0.25)

Here the *round*() function rounds to the nearest two digits to the right of the decimal point. Finally, for LL identification using the STP and FLY parameters (*LL_STP_* and *LL_FLY_*), we defined as the trailing leg the side where HC was detected.

#### 2.4.3. ORIENT: Orientation based Detection

In the hurdle clearing stride, the kinematics of the leading leg differ from those of the trailing leg [26]. Indeed, a large positive pitch angle (*θ_left_*, *θ_right_*) was expected for the leading leg during HC and large yaw angle (*ψ_left_*, *ψ_right_*) for the trailing leg. So, regardless of the IMU location (leading or trailing leg), it should always be possible to detect the HC events and determine the LL using only the pitch and yaw angles.

**Figure 6:**
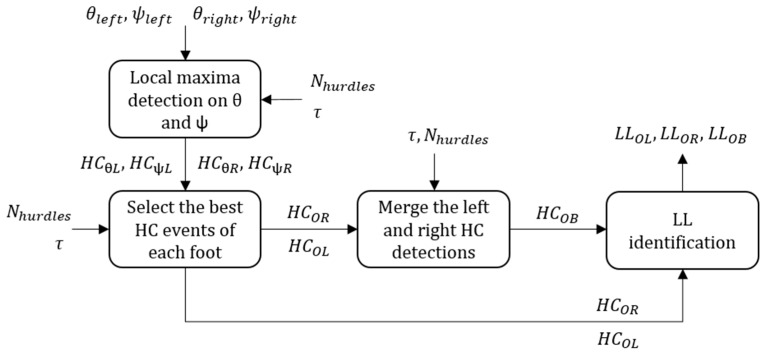
Flow chart of the ORIENT method. In the figure, the pitch and yaw signal are represented by *θ_left_*, *θ_right_* and *ψ_left_*, *ψ_right_*, respectively. *N_hurdles_* is the number of hurdles to detect and *τ* the minimum time difference between two consecutive HC. Hurdle clearance (HC) detection results are shown as *HC_XX_* and leading leg detection results as *LL_XX_*, where XX describes the detection method. For more details about the different HC and LL sets, see the definitions in Section 2.4.3.

The general behavior of the ORIENT method is depicted in Figure 6. First, this method searched for positive peaks on the pitch and yaw angles of each foot independently (unipedal). The best HC candidates obtained in unipedal configuration (*HC_OL_*, *HC_OR_*) were later combined to get the bipedal detection results (*HC_OB_*).

In unipedal configuration, we first found within each stride the local maxima on the pitch and yaw angles (i.e., separated by at least 0.5 s as for the magnetometer). We then kept the timing of the *N_hurdles_* highest peaks separated by at least *τ* (=3 s). These peaks were then stored as candidate HC events in *HC_θL_*_,_ *HC_ψL_*_,_ *HC_θR_*_,_ and *HC_ψR_* for the left foot pitch angle (*θ_left_*), left foot yaw angle (*ψ_left_*), right foot pitch angle (*θ_right_*), and right foot yaw angle (*ψ_right_*), respectively. As a result, dim(*HC_θL_*) = dim(*HC_ψL_*) = dim(*HC_θR_*) = dim(*HC_ψR_*) = *N_hurdles_*. Since an HC event produces a local maximum either on the pitch or on the yaw angle of the same foot, only *N_hurdles_* elements in *HC_θL_* ∪ *HC_ψL_* and *HC_θR_* ∪ *HC_ψR_* sets were considered as true HC events. To select the best HC candidates among all the elements in *HC_θL_* ∪ *HC_ψL_* and *HC_θR_* ∪ *HC_ψR_*, we normalized the absolute values of the peaks as in Equations (11) and (12) (only the equations for the left foot are shown):

(11) $$\hat{\theta }\left(HC_{\theta L}\right)=\left(\theta \left(HC_{\theta L}\right)-M_{\theta L}\right)/I_{\theta L}$$

(12) $$\hat{\psi }\left(HC_{\psi L}\right)=\left(\psi \left(HC_{\psi L}\right)-M_{\psi L}\right)/I_{\psi L}$$

Here, *M_θL_* is the median of the left foot pitch angle over the entire trial, *M_ψL_* the median of the yaw angle, *I_θL_* the interquartile range (IQR) of the pitch angle, and *I_ψL_* the IQR of the yaw angle. The elements in $\hat{\theta }\left(HC_{\theta L}\right)\;\cup^{\;}\;\hat{\psi }\left(HC_{\psi L}\right)$ are then sorted in descending order and added to *HC_OL_* recursively provided that each element in *HC_OL_* is separated by at least by *τ* (=3 s). As a result, we defined *HC_OL_* as the set of the HC events obtained for the left foot (i.e., the best HC candidates among *HC_θL_* and *HC_ψL_*). The same method was applied for the right foot to obtain *HC_OR_* (Figure 6). Finally, *HC_OL_* and *HC_OR_* were combined to get the bipedal detection results, with a selection process similar to the magnetometer:

(1) If |*HC_OL_(i)* – *HC_OR_(j)*| < 0.4 s, i and j ∈ {1, …, *N_hurdles_*}, then 0.5 * (*HC_OL_(i)* + *HC_OR_(j)*) was added to *HC_OB_*. Here, we assumed that if two HC events occurred within a short period (i.e., 0.4 s = average flight time in [22]) and were detected on the left and right foot distinctively, then these events were likely to correspond to a true HC. As we could not predict which of the left or right event was more accurate, we defined the time of the true HC event as the average of the left and right foot events.
  The *i* and *j* indices not considered in step 1 were recursively added to *HC_OB_* until dim(*HC_OB_*) = *N_hurdles_*. The greatest peaks were added first if they were minimum *τ* = 3 s away from all the HC already in *HC_OB_*. Finally, the results were sorted in their order of appearance within the race.

Lastly, we used the following rule to detect the LL: the leg for which an HC event corresponded to a peak in the pitch angle was labeled as the leading leg. The results were kept in three LL identification sets: *LL_OL_* and *LL_OR_* for unipedal detection of the left and right leg and *LL_OB_* for bipedal configuration.

### 2.5. Data Analysis

Ideally, the time when the athlete’s center of mass cleared the hurdle should be used as a reference for HC time. However, due to the lack of synchronization between the camera and the IMUs, this reference was not available. Instead, we considered the time of *HC_FLY_* (65% of flight phase) as the reference HC time (*HC_ref_*) if it occurred inside of the flight phase of an HC observed on video. Note that the LL at each HC was manually labeled using the video.

The HC detected using the TEMP, ORIENT, and MAG methods were considered correctly detected if it occurred inside the flight phase of a reference *HC_ref_*. Note that for *HC_STR_* and *HC_SW_* (TEMP methods in unipedal configuration), an HC was considered correctly detected if *HC_ref_* occurred inside of a stride of *HC_STR_* or inside of a swing of *HC_SW_* (Figure 5). These two exceptions were necessary as the system could not identify the LL solely based on the STR and the SW parameters. We evaluated the performance of the proposed systems by computing the mean, SD, the minimum, and the maximum number of correctly detected HC per trial. Moreover, the mean ± SD of the differences in HC detection time (Δt_HC_) between *HC_ref_* and HC detected from TEMP, ORIENT, and MAG methods were measured. Finally, all the correctly detected HC were collected, and the percentage of correctly identified LL was calculated regardless of the athlete.

As one of the goals of this study was to provide feedback to the participants and trainers, we extracted key performance features from the races [27,28], such as contact time (CT), flight time (FLY), step frequency (STF) and speed (SPE). Moreover, we obtained the speed using the distance between the hurdles (*D_H_*) and the time difference between two consecutive HC. The potential detection errors of IC and TC [18] combined with a ±10% error on the 65% reference threshold (Equation (9)) provided a confidence interval on the estimated speed. To assess how CT, STF, FLY, and SPE changed during the race, we extracted the mean of these parameters for the 11 intervals of the 15 races. We then grouped the results per interval and computed the inter-subjects mean and SD for each interval. Note that the first and last two steps of each interval were removed as these may be affected by the landing and takeoff phases. Also, we used a one-way ANOVA on the intra-interval means, with a significance level at 0.05 (*) and 0.01 (**), to assess any significant statistical differences between the second interval and the subsequent ones. The second interval was preferred to the first one as the latter was affected by the acceleration phase at the start of the race. Moreover, the intra-interval means of CT, STF, SPE, and FLY were expressed relative to the mean of the second interval (Equations (13)–(16)), and the evolution of these parameters during the race presented using boxplots:

(13) $$AvCT\left(k\right)=\frac{mean\left(CT\left(t_{k}\right)\right)}{mean\left(CT\left(t_{2}\right)\right)}$$

(14) $$AvSTF\left(k\right)=\frac{mean\left(STF\left(t_{k}\right)\right)}{mean\left(STF\left(t_{2}\right)\right)}$$

(15) $$AvSPE\left(k\right)=\frac{mean\left(SPE\left(t_{k}\right)\right)}{mean\left(SPE\left(t_{2}\right)\right)}$$

(16) $$AvFLY\left(k\right)=\frac{mean\left(FLY\left(t_{k}\right)\right)}{mean\left(FLY\left(t_{2}\right)\right)}$$

where *k* is the interval index (here *k* = 3 … 11), and t_k_ corresponds to the steps between interval *k* and *k* + 1.

## 3. Results

**Figure 7:**
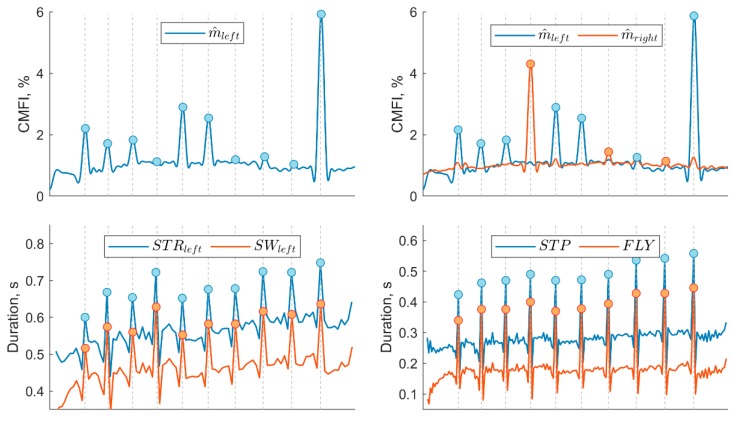

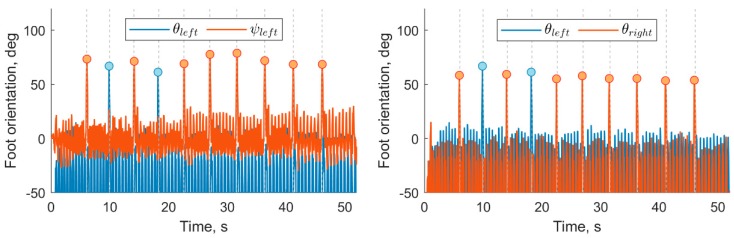
Detection results obtained by MAG (**top**), TEMP (**middle**), and ORIENT (**bottom**) methods for one trial. The magnetometer graphs show the calibrated magnetic field intensity (CMFI) in percent of the Earth’s magnetic field. The column on the left shows the detection results in unipedal configuration and on the right for bipedal detection. The vertical grey dashed lines represent the reference HC events.

In total, we analyzed the races of 15 athletes. One athlete had to be removed from the data set due to an instrumentation error. Such collection led to 300 HC for the evaluation in unipedal configuration (i.e., foot considered independently) and 150 HC for the assessment of bipedal configuration. According to the video-based validation of the HC detected in *HC_FLY_*, all the 150 HC were correctly detected, with the correct number of steps in each interval and the correct leading leg identified. The results of the MAG, TEMP, and ORIENT detection methods are compared in Figure 7, where the processed signals and detected peaks are illustrated for both unipedal and bipedal configurations.

Table 2 compares the HC detection results in terms of correctly detected HC per trial and the mean ± SD of Δt_HC_. In unipedal configuration, LL detection accuracy is shown exclusively for the ORIENT method as the other methods cannot perform such analysis. Also, LL identification and Δt_HC_ statistics were computed on the correctly detected HC, hence the different N values for each method in Table 2.

**Figure 8:**
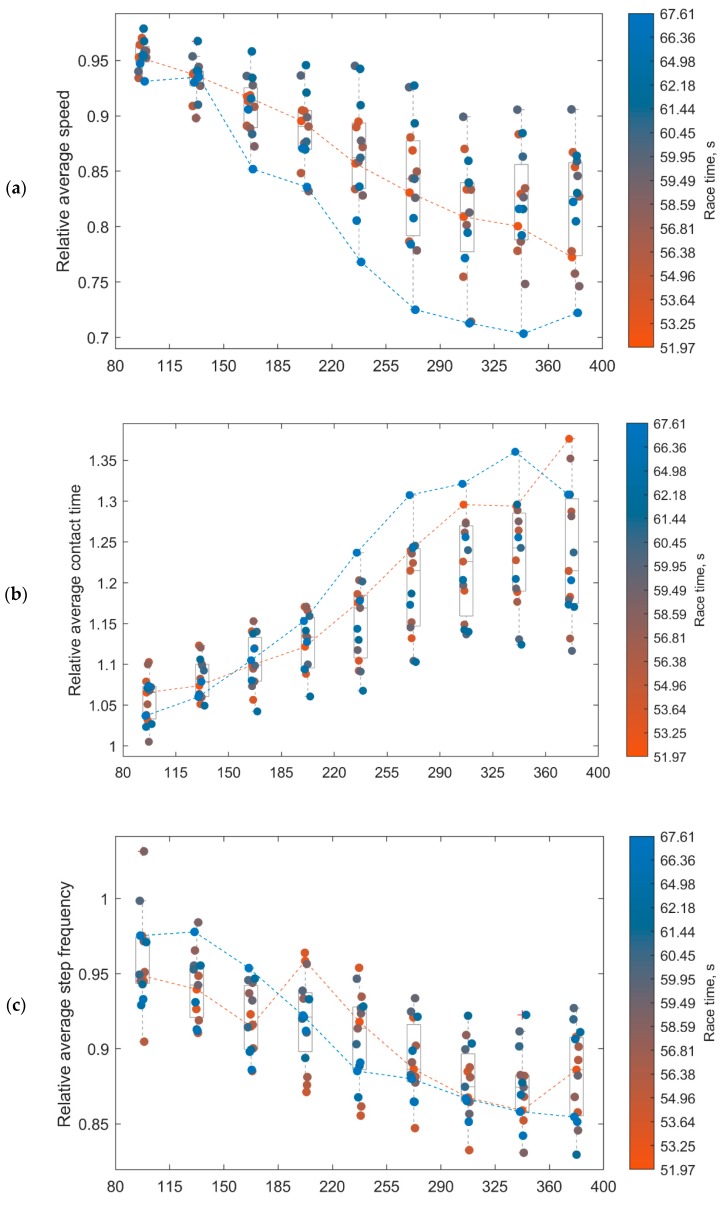

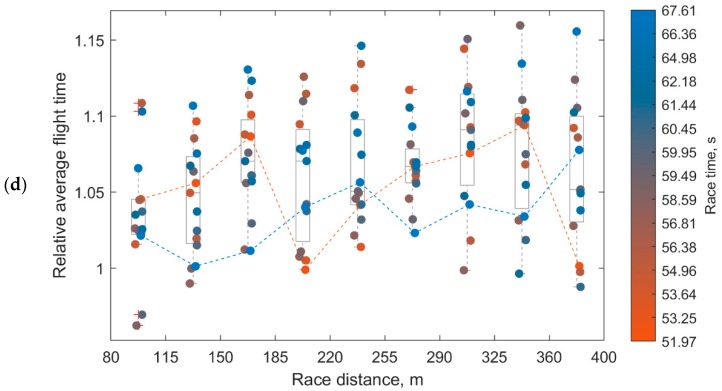
Evolution of *AvSPE* (**a**), *AvCT* (**b**), *AvSTF* (**c**), and *AvFLY* (**d**) between the third and the last interval. The y-axis values are expressed relative to the average values obtained within the second interval (45 to 80 m). The orange to blue gradient is used to differentiate the athletes according to their performance time, with the boundary performances (slowest and fastest athlete) being illustrated with dashed lines. For better visibility of individual data points, a small scatter was introduced in the x-direction.

Figure 8 presents the relative changes for the average speed (*AvSPE*), contact time (*AvCT*), step frequency (*AvSTF*), and flight time (*AvFLY*) throughout the race. These are expressed relative to the speed, contact time, step frequency, and flight time (Equations (13)–(16)) estimated in the second interval (i.e., 45–80 m). As the vertical color bar on the right side of the figures indicates, the values of the slowest athletes are shown in blue, and the fastest in orange—the boundary performances (i.e., the fastest and the slowest athlete) are shown with dashed lines.

Table 3 presents inter-subject mean and SD of the average CT, FT, STF, and SPE in each interval. The results from the one-way ANOVA test are shown for the intra-interval mean contact time, flight time, step frequency, and speed. On average, 18 steps (min = 11, max = 20) were available per interval.

**Figure 9:**
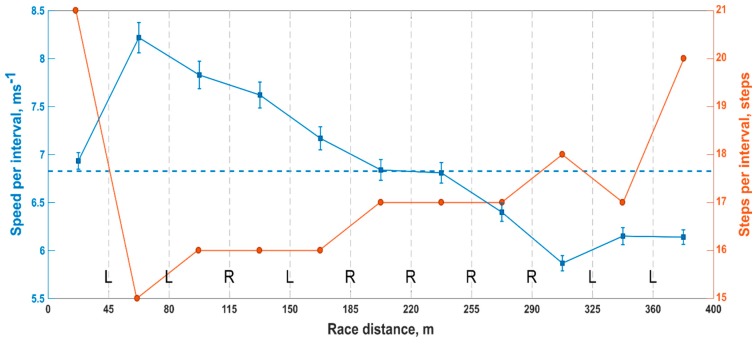
Average speed, number of steps, and leading leg analysis for each interval between hurdles within a single race. The average speed between all HC is shown with blue squares and the confidence interval with vertical solid lines. The blue horizontal dashed line corresponds to the average race speed. Also, the number of steps is depicted with orange circles and the leading leg at each HC (vertical grey dotted line) with R (right), or L (left).

Finally, Figure 9 presents an overview of the average speed (blue) and the number of steps (orange) within each HC interval of a single athlete. The blue vertical lines represent the confidence interval of the average speed values, and the blue horizontal dashed line shows the average speed during the race. Also, we used R (right) and L (left) letters to indicate the side of the leading leg at each HC (vertical grey dashed lines). Such a graph provides an example of the type of feedback that can be instantly extracted using the proposed HC detection method.

## 4. Discussion

The primary aim of this study was to evaluate the performance of three different MIMU-based methods in detecting HC events and identifying the leading leg in 400 m hurdles. In the unipedal configuration, the best HC detection results were obtained using the TEMP method and the swing phase duration (Table 2). This method was able to detect all the 300 HC available in the data set.

In contrast to the SW parameter, the ORIENT method delivered a slightly lower HC detection accuracy (95.3%), with one trial detecting only seven hurdles. The ORIENT method relies on the HC technique used by the athletes. It assumes a large pitch angle for the leading leg and a large yaw angle for trailing leg at HC (Figure 7). Its detection accuracy thus may decline the lower the performances, e.g., for beginners. However, ORIENT was the only method capable of identifying the leading leg in unipedal configuration and showed a high 99.7% accuracy with only one misclassification among all the 286 correctly detected HC.

The MAG method did not provide a reliable detection in unipedal configuration, with only 46.3% accuracy (Table 2). Closer inspection of the signals showed that most of the non-detected HC were caused by the absence of a peak in the raw data. A possible explanation for these results resides in the fact that the detectable distance between the foot-worn magnetometer and a hurdle depends on the setup of the magnet bars. As the weight of the hurdles is regulated, the number of vertically stacked magnets was limited, and so was the detectable distance. Also, fixing the magnets on the top bar of the hurdles would have reduced the foot-to-magnet distance but was not feasible in this study for practical reasons. Finally, the HC technique of the athlete may affect the detection results as an efficient technique minimizes the distance between the athlete center of mass and a hurdle. Although the results improved in the bipedal configuration (73.3%), this method remains the least accurate compared to the two others.

In the bipedal configuration, the flight time and step duration (TEMP method) provide a 100% accurate detection of HC and the ORIENT method 96% accuracy. These observations can be generalized to the identification of the leading leg in the bipedal configuration. Based on these findings, flight time seems to be the best indicator for both HC and LL identification and should be preferred to the step duration as it provides a narrower window around HC events (Figure 5). Yet, if only one foot-worn IMU is available, the swing phase duration in combination with the foot pitch and yaw angles also provides accurate detection for HC and LL.

A note of caution is due here since previous researches have shown that the vertical speed varies during the flight phase [29] and that the 65–35% ratio used in this study may change among athletes [23,24,25]. The estimated time of HC and the average speed between two hurdles must, therefore, be presented with an appropriate confidence interval (Figure 9). In future investigations, it would be interesting to investigate if: (1) the pitch angle of the leading leg and the yaw angle of the trailing leg can be used conjointly to estimate the exact moment the athlete’s center of mass clears the hurdle and (2) if instrumented magnetic hurdles with the magnet placed on the horizontal bar could be used to estimate the distance between the hurdle and the foot.

The secondary aim of this study was to investigate the evolution of contact time, running speed, flight time, and step frequency throughout the race. As shown in Figure 8 and Table 3, contact time increased, and speed decreased with the distance covered. A significant rise (*p* < 0.01) was found for contact time starting from the sixth interval in comparison to the second interval. However, the rate of these changes did not appear to be associated with the performances of the athlete as the slowest and fastest participants presented similar rates of change.

The running speed was significantly reduced as the distance covered increased, starting with the fifth interval (*p* < 0.05) and increasing (*p* < 0.01) from the sixth interval. As for the contact time, no association between the change in running speed during the race and athlete caliber was evident. These results support the evidence from previous studies [21,30], who also observed a significant increase in contact time and a decrease in running speeds for 400-m sprints. Note that the average and SD of step frequency measured in this study (3.52 ± 0.19) are comparable with those of national-level hurdlers presented in [28]. We also observed that the step frequency significantly decreased starting from the third interval.

Interestingly, flight time did not follow the same trend, and no clear pattern emerged from data analysis. Although the average flight time increased as the race progressed, only intervals 9 and 10 provided significant differences. This observation suggests that the flight time is less affected by fatigue than the contact time. However, a measure of stride length would be useful in future studies to further investigate the evolution of spatiotemporal variables as a function of fatigue during 400 m hurdle races.

Finally, Figure 9 presents the example of a report which was provided to the athletes and trainers. Such a graph showcases the potential of the proposed system and the type of feedback that can be provided during field training. Overall, this research offers new insight into the performance of different wearable methods for detecting HC and will contribute to a deeper understanding of the discipline by providing a tool for researchers, athletes, and trainers.

## 5. Conclusions

This study showed that foot-worn inertial and magnetic sensors, combined with magnets bars, can be used to detect hurdle clearing events in 400-m hurdle. The results showed that both unipedal and bipedal configuration can provide reliable detection. When the sensor is placed on one foot (unipedal configuration), the swing phase duration was capable of detecting 100% of the hurdle clearances. When combined with the pitch and yaw angles of the foot, the unipedal configuration can correctly identify the leading leg with an accuracy of 99.7%. These results were even improved to a 100% accuracy in both HC detection and leading leg identification when using flight phase duration in bipedal configuration (a sensor at each foot). Moreover, this study also showed that the use of additional magnets/magnetometer does not improve the detection results of the system. Finally, this study showcased the potential benefit of using foot-worn IMUs and validated algorithms in 400-m hurdle races as they can provide helpful feedback about the race and continuously assess the changes in spatiotemporal parameters.

## Figures and Tables

**Table 1:** Features and configurations required in order to estimate, for each step/stride, the parameters used to detect hurdle clearance.

Parameters	Detection Required		
	IC	TC	Configuration
STR	yes	no	Unipedal
SW	yes	yes	Unipedal
STP	yes	no	Bipedal
FLY	yes	yes	Bipedal

**Table 2:** Hurdle clearance and leading leg detection results for both unipedal and bipedal configurations. In total, 15 trials with each *N_hurdles_* = 10 hurdle clearances were available for the performance analysis.

Methods	HC Detection per Trial				Correct HC	Δt_HC_ (ms)		LL Accuracy
	Mean	SD	min	max	/Total HC	Mean	SD	% (Total)
**Unipedal**								
MAG	4.63	2.76	0	9	139/300	−12	100	-
TEMP_STR_	9.97	0.18	9	10	299/300	−138	106	-
TEMP_SW_	10	0	10	10	300/300	−78	104	-
ORIENT	9.53	0.82	7	10	286/300	−47	96	99.7 (285)
**Bipedal**								
MAG	7.33	1.76	2	9	110/150	15	94	39.1 (43)
TEMP_STP_	10	0	10	10	150/150	2	4	100 (150)
TEMP_FLY_	10	0	10	10	150/150	0	0	100 (150)
ORIENT	9.6	0.91	7	10	144/150	−42	33	99.3 (143)

**Table 3:** Inter-subject mean and SD of the average contact time (CT), flight time (FLY), step frequency (STF), and speed (SPE) within each interval. The results from the one-way ANOVA test, which compared the mean statistics between the second interval and the subsequent ones, are shown with significance level at 0.05 (*) and 0.01 (**).

Interval	Distance, m	CT, ms		FLY, ms		STF, Hz		SPE, ms^−1^	
		Mean	SD	Mean	SD	Mean	SD	Mean	SD
1	0–45	110	7	154	11	3.8	0.15	6.73	0.44
2	45–80	104	8	160	10	3.81	0.11	7.77	0.64
3	80–115	110	9	165	10	3.65 *	0.13	7.41	0.61
4	115–150	113	9	167	10	3.59 **	0.11	7.24	0.55
5	150–185	115	10	171	10	3.51 **	0.1	7.05 *	0.57
6	185–220	118 **	10	169	7	3.5 **	0.11	6.88 **	0.56
7	220–255	120 **	10	170	8	3.46 **	0.11	6.72 **	0.63
8	255–290	125 **	11	170	9	3.4 **	0.09	6.5 **	0.64
9	290–325	127 **	10	173 *	11	3.35 **	0.13	6.28 **	0.61
10	325–360	129 **	12	172 *	13	3.33 **	0.11	6.34 **	0.59
11	360–400	128 **	10	170	10	3.36 **	0.11	6.34 **	0.54

## Abbreviation

**Acronyms**	**Definition**
*AvCT(k)*	Average CT within the *k*th interval expressed relatively to *AvCT*(2)
*AvFLY(k)*	Average FLY within the *k*th interval expressed relatively to *AvFLY*(2)
*AvSPE(k)*	Average SPE within the *k*th interval expressed relatively to *AvSPE*(2)
*AvSTF(k)*	Average STF within the *k*th interval expressed relatively to *AvSTF*(2)
CMFI	Calibrated magnetic field intensity
CT	Contact time
*D_H_*	Distance between the hurdles
FF	Functional frame
FLY	Flight phase duration
GF	Global frame
HC	Hurdle clearance
*HC_FLY_*	HC detection results of the FLY parameter in the TEMP method
*HC_MB_*	Bipedal HC detection results of the MAG method
*HC_ML_*	Left foot HC detection results of the MAG method
*HC_MR_*	Right foot HC detection results of the MAG method
*HC_ψL_*	Left foot HC detection results based on *ψ_left_* in the ORIENT method
*HC_ψR_*	Right foot HC detection results based on *ψ_right_* in the ORIENT method
*HC_OB_*	Bipedal HC detection results of the ORIENT method (*HC_OL_* and *HC_OR_* combined)
*HC_OL_*	Left foot HC detection results of the ORIENT method (*HC_θL_* and *HC_ψL_* combined)
*HC_OR_*	Right foot HC detection results of the ORIENT method (*HC_θR_* and *HC_ψR_* combined)
*HC_ref_*	Reference HC time
*HC_STP_*	HC detection results of the STP parameter in the TEMP method
*HC_STR_*	HC detection results of the STR parameter in the TEMP method
*HC_SW_*	HC detection results of the SW parameter in the TEMP method
*HC_θL_*	Left foot HC detection results based on *θ_left_* in the ORIENT method
*HC_θR_*	Right foot HC detection results based on *θ_right_* in the ORIENT method
IC	Initial contact
IMU	Inertial measurement unit
LL	Leading leg
*LL_FLY_*	Bipedal LL detection results of the TEMP method using the FLY parameter
*LL_MB_*	Bipedal LL detection results of the MAG method
*LL_STP_*	Bipedal LL detection results of the TEMP method using the STP parameter
*m_left_*	Magnetometer signal recorded on the left foot
$\hat{m}$_*left*_	Preprocessed magnetometer signal from the left foot
*m_right_*	Magnetometer signal recorded on the right foot
$\hat{m}$_*right*_	Preprocessed magnetometer signal from the right foot
MAG	Magnetometer based method for HC and LL detection
MIMU	Magnetic inertial measurement unit
MS	Mid-stance
*N_hurdles_*	Total number of hurdles
ORIENT	Orientation based method for HC and LL detection
*ψ*	Yaw angle
$\hat{\psi }$	Normalized yaw angle
*ψ_left_*	Yaw angle measured on the left foot
*ψ_right_*	Yaw angle measured on the right foot
SPE	Speed
STF	Step frequency
STP	Step duration
STR	Stride time
SW	Swing phase duration
*τ*	Minimum time difference between two consecutive HC
*T_race_*	Official race time of a participant
*t_start_*	Time of the start of the race
Δt_HC_	Differences in HC detection time
TC	Terminal contact
TEMP	Temporal parameter-based method for HC and LL detection
*θ*	Pitch angle
$\hat{\theta }$	Normalized pitch angle
*θ_left_*	Pitch angle measured on the left foot
*θ_right_*	Pitch angle measured on the right foot
*V_max_*	Maximum running speed considered (42 km/h)
